# Investigation of optical coherence micro-elastography as a method to visualize micro-architecture in human axillary lymph nodes

**DOI:** 10.1186/s12885-016-2911-z

**Published:** 2016-11-09

**Authors:** Kelsey M. Kennedy, Lixin Chin, Philip Wijesinghe, Robert A. McLaughlin, Bruce Latham, David D. Sampson, Christobel M. Saunders, Brendan F. Kennedy

**Affiliations:** 1 Optical+Biomedical Engineering Laboratory, School of Electrical, Electronic & Computer Engineering, The University of Western Australia, 35 Stirling Highway, Perth, WA 6009 Australia; 2 BRITElab, Harry Perkins Institute of Medical Research, QEII Medical Centre, 6 Verdun St, Nedlands, Perth, WA 6009 Australia; 3 Australian Research Council Centre of Excellence for Nanoscale BioPhotonics, School of Medicine, Faculty of Health Sciences, University of Adelaide, Adelaide, SA 5005 Australia; 4 PathWest, Fiona Stanley Hospital, Robin Warren Drive, Murdoch, WA 6150 Australia; 5 Centre for Microscopy, Characterisation & Analysis, The University of Western Australia, 35 Stirling Highway, Perth, WA 6009 Australia; 6 School of Surgery, The University of Western Australia, 35 Stirling Highway, Perth, WA 6009 Australia; 7 Breast Clinic, Royal Perth Hospital, 197 Wellington Street, Perth, WA 6000 Australia

**Keywords:** Breast cancer, Lymph node, Optical coherence tomography, Elastography, Mechanical properties, Intraoperative

## Abstract

**Background:**

Evaluation of lymph node involvement is an important factor in detecting metastasis and deciding whether to perform axillary lymph node dissection (ALND) in breast cancer surgery. As ALND is associated with potentially severe long term morbidity, the accuracy of lymph node assessment is imperative in avoiding unnecessary ALND. The mechanical properties of malignant lymph nodes are often distinct from those of normal nodes. A method to image the micro-scale mechanical properties of lymph nodes could, thus, provide diagnostic information to aid in the assessment of lymph node involvement in metastatic cancer. In this study, we scan axillary lymph nodes, freshly excised from breast cancer patients, with optical coherence micro-elastography (OCME), a method of imaging micro-scale mechanical strain, to assess its potential for the intraoperative assessment of lymph node involvement.

**Methods:**

Twenty-six fresh, unstained lymph nodes were imaged from 15 patients undergoing mastectomy or breast-conserving surgery with axillary clearance. Lymph node specimens were bisected to allow imaging of the internal face of each node. Co-located OCME and optical coherence tomography (OCT) scans were taken of each sample, and the results compared to standard post-operative hematoxylin-and-eosin-stained histology.

**Results:**

The optical backscattering signal provided by OCT alone may not provide reliable differentiation by inspection between benign and malignant lymphoid tissue. Alternatively, OCME highlights local changes in tissue strain that correspond to malignancy and are distinct from strain patterns in benign lymphoid tissue. The mechanical contrast provided by OCME complements the optical contrast provided by OCT and aids in the differentiation of malignant tumor from uninvolved lymphoid tissue.

**Conclusion:**

The combination of OCME and OCT images represents a promising method for the identification of malignant lymphoid tissue. This method shows potential to provide intraoperative assessment of lymph node involvement, thus, preventing unnecessary removal of uninvolved tissues and improving patient outcomes.

## Background

The presence of lymph node metastasis is one of the primary prognostic indicators for patients with early-stage breast cancer [1–3]. During surgery, the sentinel lymph node (SLN) (the first-draining node) is often excised and assessed. If the SLN is found to be involved, axillary lymph node dissection (ALND) is often performed to determine if the disease is present in the lymphatic system and to prevent its further spread [3]. However, ALND is associated with potentially significant long term morbidity, such as seroma formation, altered sensation in the upper limb, and lymphedema [4]. The accuracy of SLN assessment is, thus, imperative in avoiding unnecessary ALND. Current pre- and intra-operative SLN assessment techniques include macroscopic examination, pathology, and medical imaging [5]. Macroscopic assessment is subjective; for example, when performing visual inspection and palpation alone, inflammation in nodes may be mistaken for malignancy [6]. Intraoperative pathology techniques, including frozen section and imprint cytology, provide high-resolution, highly specific assessment of lymph node morphology, but are often expensive, time-consuming (20–40 min), and can sample only a small proportion of the lymph node within an intraoperative timeframe [7]. This can result in low diagnostic accuracy: the sensitivities of frozen section and imprint cytology have been reported to be in the range 53–74 %, with corresponding specificities of 98–100 % [8, 9]. Medical imaging, including magnetic resonance imaging (MRI) [10], ultrasound (US) [11], and positron emission tomography/X-ray computed tomography (PET-CT) [12], have also been proposed for preoperative staging of the axilla. These techniques rely predominantly on assessment of the size and shape of the nodes, as they do not typically have the resolution required to visualize micro-scale lymph node morphology, limiting their potential for SLN assessment. For example, these medical imaging techniques can have difficulty in distinguishing metastatic and reactive nodes [11].

New techniques for rapid, accurate, intraoperative assessment of the SLN have the potential to improve surgical decision-making, ultimately leading to improved patient outcomes. Methods such as high-frequency ultrasound (HF-US) [13] and photoacoustic tomography (PAT) [14] have been investigated, reporting spatial resolutions of ~100 μm (HF-US) or ~50 μm (PAT); additionally, PAT has been used in conjunction with external contrast agents. To obtain improved spatial resolution without the need for labelling agents, optical modalities, such as optical coherence tomography (OCT), often described as the optical analog to ultrasonography, have been proposed [15–22]. The detection of backscattered light, rather than sound, provides OCT with a spatial resolution of 1–20 μm, but only to a depth of 1–2 mm in turbid tissue. OCT has the potential to rapidly scan entire ex vivo lymph nodes intraoperatively, improving upon the sampling issues of intraoperative histopathology techniques. However, it is unclear if lymph node metastases can be adequately delineated based on endogenous optical scattering contrast alone [21, 22].

It has previously been established that the mechanical properties of malignant lymph nodes are often distinct to those of normal nodes [23, 24]. Mapping the micro-scale mechanical properties of lymph nodes could, thus, provide additional diagnostic information to complement that provided by OCT. Over the past 25 years, various imaging techniques, collectively termed elastography, have emerged to map the mechanical properties of tissues. Elastography uses an imaging modality, most commonly US [25] or MRI [26], to measure tissue deformation in response to a mechanical load. Tissue mechanical properties are then estimated using a mechanical model, and mapped onto an image, known as an elastogram. Several studies have investigated the use of US elastography to assess axillary lymph nodes [27, 28]. However, the spatial resolution of elastography is ultimately limited by the underlying imaging modality, which, using US or MRI, may be insufficient to detect lymph node metastasis.

Optical coherence elastography (OCE) [29–32] uses OCT as the underlying modality and, thus, has a higher spatial resolution than US- or MRI-based elastography [30]. Our group has recently demonstrated a variant of OCE, dubbed optical coherence micro-elastography (OCME), which has a lateral resolution matched to that of OCT over *en face* fields of view up to ~20 × 20 mm [33]. In OCME, a uniform compressive load is applied to the specimen, and 3-D elastograms are generated of the resulting mechanical strain, which is inversely proportional to the tissue stiffness. We have shown that this technique can provide contrast between malignant and normal tissue in fresh, excised human breast specimens [33, 34].

Here, we utilized OCME to scan axillary lymph nodes, freshly excised from human breast cancer patients, to investigate its potential for the intraoperative assessment of lymph nodes. We imaged 26 specimens from 15 patients undergoing a mastectomy or breast-conserving surgery with axillary clearance and present co-located histology, OCT images, and elastograms of selected representative cases. The results show that OCME provides contrast that is additional and complementary to that provided by OCT and demonstrate the potential of OCME for intraoperative SLN assessment.

## Methods

### Imaging system

**Fig. 1:**
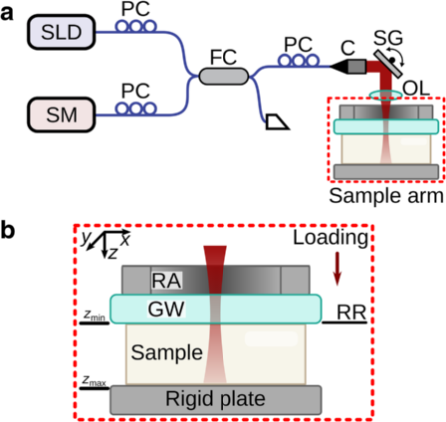
**a** Schematic of the imaging system used in this study. Collimator (C), 50/50 fiber coupler (FC), objective lens (OL), polarization controller (PC), scanning galvanometer (SG), superluminescent diode light source (SLD), spectrometer (SM). **b** Schematic (*side view*) of the sample stage, corresponding to the region within the *red box* in (**a**). Glass window (GW), piezoelectric ring actuator (RA), reference reflector (RR)

A portable OCE system, described in detail previously [33, 34], was used in this study. The system utilizes an 835-nm, spectrometer-based OCT system, with axial/lateral resolutions of 8 μm/11 μm (in air), respectively. 3-D datasets were acquired in ~17 min, with dimensions (*x* × *y* × *z*) up to 10 × 10 × 1.5 mm, comprising 1000 line scans (A-scans) in each 2-D image (B-scan) and 10,000 B-scans in each volume (C-scan). An annular piezoelectric transducer (Piezomechanik, Germany) with a glass window fixed to the surface was used to enable imaging and mechanical loading from the same side of the sample [33, 34]. Figure 1 shows a schematic diagram of this imaging system.

### Imaging protocol

To ensure even contact with the window, the specimen was preloaded prior to imaging by applying ~10–20 % compression. During imaging, the transducer displaced the tissue surface by up to a maximum of ~2 μm, at a frequency of 5 Hz, and was synchronized with the OCT acquisition such that consecutive B-scans were acquired in the unloaded then loaded state. The local axial tissue displacement was proportional to the phase difference between corresponding A-scans in consecutive B-scans, as measured using phase-sensitive OCT [35]. Five pairs of B-scans were recorded and averaged at each *y*-position to improve the precision of the measured displacement [33]. A phase-unwrapping algorithm, described previously, was used to improve the dynamic range of measurable displacements [33]. Elastograms were generated from the displacement maps by calculating the local strain (change in displacement assessed over an axial depth range of 100 μm, which defined the elastogram axial resolution) throughout the sample using a weighted least-squares linear regression algorithm [33]. The lateral (*x,y*) resolution of the elastograms matched that of the OCT system (11 μm). All datasets were processed using custom software implemented in Matlab (Mathworks, USA, v2012b).

OCT is effective in identifying adipose, which exhibits optical scattering patterns distinct from those of other tissues. However, the low OCT signal within adipose cells introduces significant noise into the elastograms. Adipose, and other areas of low OCT signal, were manually segmented from the elastograms using image processing software (GNU Image Manipulation Program, v2.8.14), and the remaining OCME data (elastogram) overlaid onto the OCT image [34]. Elastograms are presented on a linear false-color scale in millistrain (mε), i.e., length change per unit length × 10^−3^, and OCT images are presented on a logarithmic decibel (dB) grayscale. As the amount of contrast provided by OCME is dependent on the sample structure and applied load [34] (see Discussion), the color-scale of the presented elastograms was chosen to maximize the available image contrast. The presented *en face* OCT images and elastograms are taken from within 300 μm of the sample surface, chosen as the *en face* images that best matched the corresponding histology.

### Tissue preparation

Informed consent was obtained from patients and the study approved by the Human Ethics Committee of Sir Charles Gairdner Hospital, Perth, Western Australia. Twenty-six specimens were imaged from 15 patients undergoing a mastectomy or breast-conserving surgery with axillary clearance. Samples were bisected to allow imaging of the internal face of each node and kept hydrated in saline until imaging, which occurred within 1–2 h of excision. After imaging, specimens were fixed in 10 % neutral buffered formalin, embedded in paraffin, sectioned and stained with hematoxylin and eosin (H&E), following standard histopathology protocols. The H&E-stained sections were digitally micrographed (ScanScope, Leica Biosystems, Nussloch, Germany) and manually co-registered with the corresponding *en face* elastograms and *en face* OCT images. Interpretation of histology was performed by an experienced pathologist (B. Latham).

## Results

**Fig. 2:**
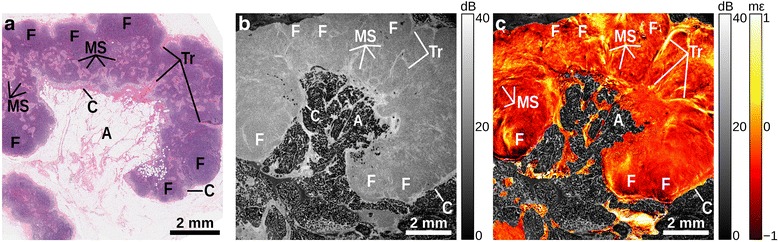
Benign lymph node sample. **a** H&E-stained histology, (**b**) *en face* OCT image, and (**c**) elastogram overlaid on OCT. Adipose (A), capsule (C), follicle (F), medullary sinuses (MS), trabeculae (Tr)

Figure 2 shows an example of a normal, benign lymph node. The histology (Fig. 2a) shows that the node has a fibrous capsule (C) separating the lymphoid tissue from surrounding adipose (A). Several follicles (F) comprising sheets of lymphocytes are observed in the cortex of the node. Below the cortex, the medulla comprises medullary cords separated by medullary sinuses (MS). Fibrous trabeculae (Tr) are also visible. In the OCT image (Fig. 2b), the fibrous capsule is distinguished by higher backscattering, and the surrounding adipose provides a distinctive honeycomb structure. Thin strands of high backscattering, corresponding to collagen fibers in the trabeculae and medullary sinuses, are present throughout the node. In the elastogram (Fig. 2c), the capsule is identified by an abrupt change in strain. The elastogram provides additional contrast of trabeculae compared to the OCT. The follicles present as oval-shaped regions that are separated by changes in strain caused by surrounding trabeculae and sinuses. Smaller, irregularly shaped local areas of greater negative (compressive) strain in the medulla correspond to medullary sinuses.

**Fig. 3:**
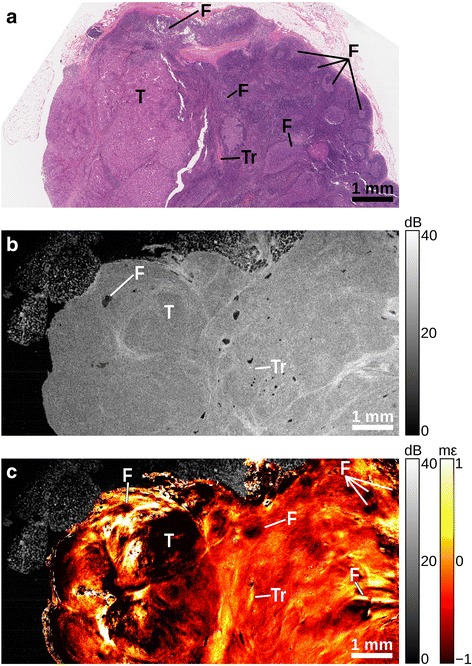
Lymph node containing metastasis. **a** H&E-stained histology, (**b**) *en face* OCT image, and (**c**) elastogram overlaid on OCT. Follicle (F), tumor (T), trabeculae (Tr)

Figure 3 shows a lymph node containing a ~3 mm-diameter region of metastatic tumor (T). The remainder of the sample is normal lymphoid tissue and contains several follicles (F) with relatively large germinal centers. Fibrous trabeculae (Tr) are also observed running through the normal portion of the node. The OCT image (Fig. 3b) highlights areas of collagen, including the trabeculae, and some follicles are also distinguishable. The OCT image does not, however, provide contrast between the normal and malignant regions. On the other hand, the tumor is readily differentiated from normal tissue in the elastogram (Fig. 3c). Strain heterogeneity within the malignant region is attributed to groups of tumor cells separated by fibrous bands. Within the normal region of the lymph node, pockets of high negative strain correspond to germinal centers within follicles.

**Fig. 4:**
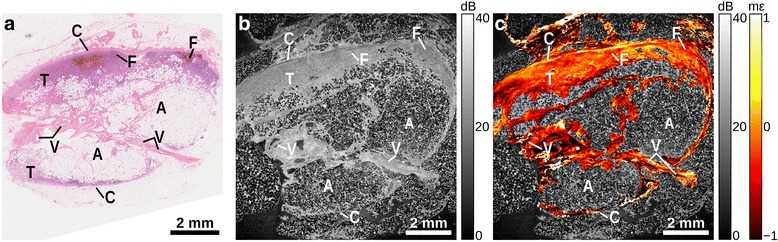
Lymph node containing pleomorphic lobular carcinoma. **a** H&E-stained histology, (**b**) *en face* OCT image, and (**c**) elastogram overlaid on OCT. Adipose (A), capsule (C), follicle (F), tumor (T), vessel (V)

Figure 4 shows a lymph node containing a ~3 × 1 mm region of metastatic tumor (T) classified as pleomorphic lobular carcinoma. The top left of the node contains mainly sheets of tumor cells, and these become gradually more interspersed with lymphocytes and macrophages toward the right of the sample. The far right of the sample is normal lymphoid tissue and contains follicles (F). The central region consists of a band of stroma, containing several vessels (V), surrounded by adipose (A). The OCT image (Fig. 4b) again exhibits higher backscattering in the areas of dense stroma, including the capsule (C), but presents an otherwise homogeneous signal. The elastogram (Fig. 4c) reveals a region of high negative strain at the far left corresponding to a region of solid tumor cells delineated by stroma. The elastogram is also highly heterogeneous in the central region of stroma, particularly around vessels. The remainder of the region of solid tumor cells presents as mostly homogeneous strain. The far right of the sample, corresponding to normal lymph node, contains small, local areas of negative strain, more similar to the heterogeneity seen in areas of normal lymph node in Figs. 2 and 3.

**Fig. 5:**
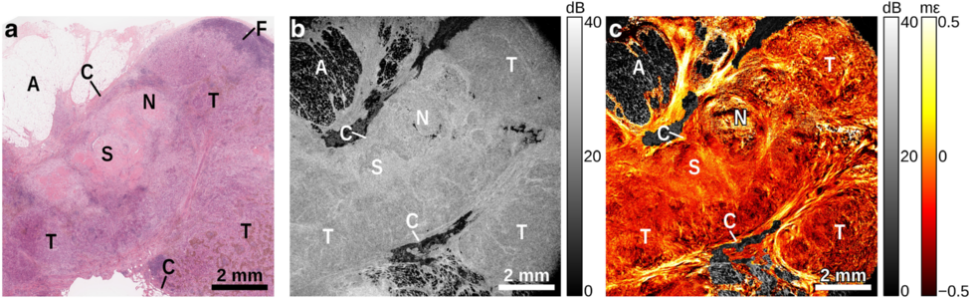
Lymph node largely replaced by metastasis. **a** H&E-stained histology, (**b**) *en face* OCT image, and (**c**) elastogram overlaid on OCT. Adipose (A), capsule (C), necrosis (N), stroma (S), tumor (T)

Figure 5 shows a region of a lymph node in which normal tissue has been largely replaced by malignancy. The histology reveals an area of fibrotic stroma (S) toward the center of the sample, with an area of necrosis (N) present, surrounded by tumor interspersed with stroma (T). In the OCT image (Fig. 5b), the capsule (C), as well as the region of fibrosis, can be identified as regions of higher backscattering. The elastogram (Fig. 5c) provides much higher contrast between the capsule and the stroma than OCT. The region of fibrosis presents as homogeneous strain, indicating its mechanical uniformity. In areas where tumor cells are interspersed with desmoplastic stroma, heterogeneous strain is present.

## Discussion

In this study, we have presented representative elastograms, with corresponding OCT and histology, selected from 26 fresh, unstained specimens. In benign and involved lymph nodes, we observed features such as adipose, capsule, follicles, and trabeculae. As with our previous OCME studies on human breast tissue [33, 34], the results show that OCME provides additional contrast that is complementary to that provided by OCT alone. This complementary information is presented using image fusion to combine the *en face* elastograms with the OCT images, in a similar manner to that employed by existing medical imaging techniques, such as MRI and PET-CT [36]. The contrast observed in the elastograms consists of both negative (compressive) strain, as expected, and also positive (tensile or compressive in the direction opposed to the applied force) strain. This accentuates feature contrast, and is a reflection of the structural heterogeneity of the tissue [34].

In the OCT images (Part b, Figs. 2, 3, 4 and 5), adipose is readily distinguished by its distinct ‘honeycomb’ appearance. The capsule surrounding the lymph node can be seen as a band of higher backscatter in OCT, and as abrupt changes in strain in OCME. Follicles make up much of the structure of healthy lymph nodes (Fig. 2), with the trabeculae between the follicles visible as bands of higher backscattering (OCT, Fig. 2b), and discontinuities in the strain pattern (OCME, Fig. 2c). Regions of tumor typically appear in the OCT images as areas of homogeneous signal, with few distinguishing features (Part b, Figs. 3, 4 and 5). The same regions can often be distinguished in the elastograms (Part c, Figs. 3, 4 and 5), exhibiting strain patterns distinct from that of uninvolved follicles (Fig. 2c). Consistent with our previous studies in breast tissue, regions of solid tumor cells result in homogeneous strain patterns; whereas, regions containing tumor mixed with desmoplastic stroma result in heterogeneous strain [34]. On the cellular level, tumor cells are often softer than the collagen-rich stroma [37, 38], which is likely the cause of the heterogeneous strain patterns observed in these mixed regions. Although the appearance of tumor varies notably from specimen to specimen, in all the cases we examined the strain patterns can be linked to the underlying micro-scale tissue constituents and architecture and, in most cases, the mechanical contrast in regions of malignancy improves upon that provided by OCT alone. This is most noticeable in Fig. 5, where the fine structure seen in the OCME of the tumor on the right of the image is absent from the OCT images. Further study is required to ascertain the extent to which these visual features are consistent over the larger population. However, we have found that the mechanical contrast provided by OCME complements the optical contrast provided by OCT, and aids in the distinction of malignant from benign lymph node tissue.

Previous studies in assessing lymph node involvement using OCT imaging alone have reported sensitivity/specificities of 92 %/83 % by Grieve *et al*. [21], and 59 %/81 % by Nolan *et al*. [22]. Grieve *et al*.’s results demonstrate the benefits of using high resolution OCT (~1 μm, approaching the ~0.5 μm of typically histology). However, this high resolution is achieved at the expense of a low depth of field (DOF, ~10 μm, using a white light source and a water immersion lens with a numerical aperture of 0.3) [21], compared to the DOF of ~0.5 mm of standard OCT (near-infrared light, lens numerical aperture of ~0.03–0.04). Low DOF typically implies that either the sample or the imaging lens must be physically moved in order to acquire in-focus images from multiple depths, slowing acquisition of 3-D volumes. Nolan *et al*. used an OCT system with comparable resolution to that used in this study (~10 μm) [22], and their results support our findings that OCT alone may not be sufficient in reliably detecting lymph node metastases. Nolan *et al*. additionally examined multiple slices through the volume in order to arrive at a diagnosis; whereas, our study analyzed a single OCT/OCME *en face* image for each sample, chosen from the depth that best matched the histology section. Full 3-D examination may potentially allow for improved diagnosis within a short time frame, and further study is required to assess the information available from the full 3-D OCME dataset.

The images presented in this study were obtained from within 300 μm of the bisected surface of the lymph nodes. The depth penetration of our OCT system in human lymphoid tissue was observed to be approximately 500–700 μm; however, the image quality of both OCT and OCME notably degrades due to attenuation of the light beam by scattering as we approach this maximum depth. Bisecting the nodes, as in this study, allows scanning of two internal faces and effectively doubles the depth information obtained. In future intraoperative deployment, both the internal and external faces of bisected nodes could be scanned, which we estimate would allow sampling of >50 % of the volume of most lymph nodes, much greater than that achievable using intraoperative histopathology [39]. The percentage of the lymph node that should be sampled for OCME to accurately judge involvement status will form part of a future study aimed at assessing the sensitivity and specificity of the technique.

Measurement of local strain in OCME is coupled to the underlying sample structure and elasticity, and to the applied load [30, 33, 34]. Related OCE techniques have been demonstrated that directly estimate elasticity based on measurement of the propagation velocity of elastic waves [30–32, 40–42]. However, these techniques have yet to overcome the challenges necessary to achieve spatial resolutions comparable with compression OCE techniques, such as OCME [30–32]. We have recently shown an adaptation of OCME that determines elasticity at the surface of tissue, using a compliant silicone layer with known mechanical properties to account for applied load [43]. However, the ability to characterize the elasticity distribution at depth in heterogeneous tissues is still to be fully investigated. Another possible solution is to solve for the elasticity distribution using an iterative computational method [44]. In addition, semi-quantitative assessment may be possible using a visual grading scale, relating features seen in OCT and OCME with the presence and type/grade of tumor.

The results of this study demonstrate that OCME can visualize multiple salient features of the micro-architecture of malignant and benign nodes, providing justification for a larger study, incorporating more specimens and patients, in order to determine the diagnostic accuracy of the technique (sensitivity and specificity). Additional study is also required to determine the minimum metastasis size that can be reliably identified with OCME. We hypothesize that this size will be dependent on the differences between the mechanical properties of the particular tumor type and normal lymphoid tissue. This lower bound is likely to be larger than the smallest metastasis detectable using histology, as the lateral resolution of OCME (~10 μm) is much coarser than that of the optical microscopes used for histology (<0.5 μm). However, we have recently demonstrated a variant of OCME with a lateral resolution of 2 μm [45] that may be able to detect micrometastases of a similar size to those detectable with histology, and further improvements may be possible by using an OCT system with even higher resolution (<1 μm) [21]. Future studies will also aim to further develop OCME toward providing results within intraoperative timeframes, which will require significant increases to acquisition and processing speeds. The current study acquired 3-D volumes in ~17 min; however, we have previously demonstrated a method of acquiring OCME volumes in ~5 s [46], which could be applied to scanning lymph nodes. Similarly, using the current software, each dataset required approximately 1 h to process on a standard PC. We have recently shown the use of custom software running on a graphics processing unit to decrease this time to under 50 s for datasets of the size recorded in this study [47]; however, further research is required to ensure the image quality matches that of the results shown here.

## Conclusion

OCME represents a new method for the identification of malignant tissue in lymph nodes, with the potential to be used intraoperatively. In this initial study, we have demonstrated the correspondence between elastograms and co-registered histology, as well as the complementary information provided by OCT and OCME. These results show the potential for future development of OCME as an intraoperative tool for assessment of lymph node involvement.

## Abbreviations

ALND: Axillary lymph node dissection
H&E: Hematoxylin and eosin
MRI: Magnetic resonance imaging
OCE: Optical coherence elastography
OCME: Optical coherence micro-elastography
OCT: Optical coherence tomography
PAT: Photoacoustic tomography
PET-CT: Positron emission tomography/X-ray computed tomography
SLN: Sentinel lymph node
US: Ultrasound
